# Germ-Free Mice Exhibit Mast Cells With Impaired Functionality and Gut Homing and Do Not Develop Food Allergy

**DOI:** 10.3389/fimmu.2019.00205

**Published:** 2019-02-12

**Authors:** Martin Schwarzer, Petra Hermanova, Dagmar Srutkova, Jaroslav Golias, Tomas Hudcovic, Christian Zwicker, Marek Sinkora, Johnnie Akgün, Ursula Wiedermann, Ludmila Tuckova, Hana Kozakova, Irma Schabussova

**Affiliations:** ^1^Laboratory of Gnotobiology, Institute of Microbiology of the Czech Academy of Sciences, Novy Hradek, Czechia; ^2^Laboratory of Cellular and Molecular Immunology, Institute of Microbiology of the Czech Academy of Sciences, Prague, Czechia; ^3^Institute of Specific Prophylaxis and Tropical Medicine, Medical University of Vienna, Vienna, Austria

**Keywords:** germ-free, mouse models, food allergy, mast cells, commensal bacteria

## Abstract

**Background:** Mucosal mast cells (MC) are key players in IgE-mediated food allergy (FA). The evidence on the interaction between gut microbiota, MC and susceptibility to FA is contradictory.

**Objective:** We tested the hypothesis that commensal bacteria are essential for MC migration to the gut and their maturation impacting the susceptibility to FA.

**Methods:** The development and severity of FA symptoms was studied in sensitized germ-free (GF), conventional (CV), and mice mono-colonized with *L. plantarum* WCFS1 or co-housed with CV mice. MC were phenotypically and functionally characterized.

**Results:** Systemic sensitization and oral challenge of GF mice with ovalbumin led to increased levels of specific IgE in serum compared to CV mice. Remarkably, despite the high levels of sensitization, GF mice did not develop diarrhea or anaphylactic hypothermia, common symptoms of FA. In the gut, GF mice expressed low levels of the MC tissue-homing markers CXCL1 and CXCL2, and harbored fewer MC which exhibited lower levels of MC protease-1 after challenge. Additionally, MC in GF mice were less mature as confirmed by flow-cytometry and their functionality was impaired as shown by reduced edema formation after injection of degranulation-provoking compound 48/80. Co-housing of GF mice with CV mice fully restored their susceptibility to develop FA. However, this did not occur when mice were mono-colonized with *L. plantarum*.

**Conclusion:** Our results demonstrate that microbiota-induced maturation and gut-homing of MC is a critical step for the development of symptoms of experimental FA. This new mechanistic insight into microbiota-MC-FA axis can be exploited in the prevention and treatment of FA in humans.

## Introduction

Food allergy (FA) is a widespread pathological immune reaction which is initiated by generally harmless food antigens. Its global prevalence has been increasing since the 1960s, especially in industrialized countries, suggesting environmental factors play a key role in the susceptibility and etiology of this disorder (1). IgE-mediated FA, which is the most common form of FA, is based on two phases: (i) allergic sensitization and (ii) the effector phase (1, 2). The humoral and cellular immune responses are clearly biased toward a type 2-related phenotype, characterized by the production of specific IgE antibodies and cytokines, such as IL-4, IL-5, IL-13, or IL-10 (3). In the effector phase, allergen-induced crosslinking of IgE bound to mast cells (MC) leads to release of histamine, serotonin and MC proteases as well as cytokines (e.g., TNF-α), resulting in the rapid appearance of symptoms, such as diarrhea and hypothermia (4). Even in individuals with high levels of food allergen-specific serum IgE, the susceptibility to develope these clinical symptoms differs dramatically (5, 6). The mechanisms underlying this phenomenon remain unknown.

The hygiene hypothesis suggests that changes in the commensal microbiota composition and/or function, due to excessive antibiotic use or increased hygiene, can increase the level of allergic sensitization (7, 8). By using germ-free (GF) animals, several studies have shown that the lack of bacteria leads to increased levels of serum IgE in comparison to colonized conventional (CV) mice (9, 10). Interestingly, colonization of GF mice with a single bacterial strain, with a mixture of several strains, or with the microbiota of CV mice through co-housing prevented the development of allergic sensitization and led to decrease of allergen-specific IgE (10–12).

*Lactobacillus plantarum* is an extremely versatile lactic acid bacterium that has been isolated from a variety of habitats, such as plants, the gastro-intestinal tracts of human and animals as well as raw or fermented dairy products (13). The human isolate *L. plantarum* WCFS1 possesses strong immunomodulatory properties, and has been shown to induce maturation of immune cells *in vitro* (14, 15) and interact with the host immune system *in vivo* (16). Specifically, oral application of *L. plantarum* WCFS1 enhanced activation of intestinal cells and shifted the Th1/Th2 balance toward a Th2 response (17). In a mouse model of peanut allergy, oral supplementation of this strain aggravated the allergic responses associated with increased MC degranulation (14).

MC are innate immune cells which are involved both in the immunological homeostasis as well as in parasitic infection (18–20) and various immunological disorders (21, 22). MC originate from CD34+ progenitors in the bone marrow and then enter the circulation and peripheral tissues, where they undergo maturation (23, 24). Being at the mucosal sites, MC are in close contact with the microbiota. Indeed, commensal bacteria have been shown to modulate several phenotypic and functional characteristics of MC, including their recruitment to the tissue, maturation and survival (23, 25). Along these lines, Kunii et al. have shown that the microbiota is required for the migration of MC to the intestine through the induction of CXCR2 ligands (23). Similarly, in the skin, the microbiota is crucial for recruitment and maturation of dermal MC (25).

Although only low numbers of MC are found in the intestine of naïve mice (26), their numbers increase in food allergy (27). The crucial role of MC in FA has been well-established (27, 28). After MC depletion with anti-c-kit antibody, CV mice do not develop OVA-induced gastrointestinal manifestation (27) and MC are also essential for the full development of hypothermia in the OVA FA mouse model (29). Additionally, transgenic mice with increased numbers of intestinal MC exhibit augmented severity of FA symptoms (30).

The literature on the interaction between microbiota, MC and susceptibility to FA is contradictory. On one hand, it has been demonstrated that GF mice exhibit altered functionality of MC and their impaired migration into the intestinal and skin tissue (23, 25). On the other hand; different studies have shown that GF mice are more susceptible to develop clinical symptoms of FA (10, 31).

In this study we seek to determine the role of commensal bacteria in the induction of FA using GF mice. We observed that GF mice did not develop the clinical symptoms of FA, such as allergic diarrhea and hypothermia, despite having higher titers of allergen-specific Th2-associated antibodies. Furthermore, the lack of commensals resulted in reduced numbers of tissue MC with low maturation status. Importantly, conventionalization of GF mice with complex microbiota through co-housing with CV mice, but not mono-colonization with *L. plantarum* WCFS1, fully recapitulated the FA phenotype observed in the CV mice. These results implicate that signals from complex microbiota are necessary for the homing of MC into the intestinal tissue as well as their maturation, which are prerequisites for developing the clinical symptoms of FA.

## Methods

### Animals

Germ-free (GF) BALB/c mice were derived from the conventional BALB/c mice by Cesarean section and kept under axenic conditions in Trexler-type plastic isolators for at least 5 generations. The sterility was controlled as previously described (32). Briefly, sterility was assessed every 2-weeks by confirming the absence of bacteria, molds, and yeast by aerobic and anaerobic cultivation of mouse feces and swabs from the isolators in VL (Viande-Levure), Sabouraud-dextrose and meat-peptone broth and subsequent plating, and aerobic/anaerobic cultivation on blood, Sabouraud and VL agar plates. Conventional (CV) *Helicobacter*-free mice were housed in individually ventilated cages (Tecniplast S.P.A., IT). Experimental animals were obtained by mating of female and male BALB/c mice and their female offspring were weaned at day 28 postnatally. Female offspring were co-housed together until day 60, when they were assigned either to the CV/OVA or CV/Ctrl group, which were housed separately thereafter. Ex-germ-free (exGF) mice were obtained by co-housing of 28-day old GF mice with age- and gender-matched CV mice. GF mice were mono-associated with *Lactobacillus plantarum* WCFS1 (Lp) and the level of bacterial colonization was evaluated weekly by plating serial dilution of feces on de Man, Rogosa, and Sharpe (MRS, Oxoid, UK) agar plates as described previously (33). Colonization remained stable throughout the experiment and reached levels of 2–3 × 10^9^ CFU/g feces. Ceca from control CV, GF, exGF, and Lp mice were weighed and a picture was taken. Cecum content was frozen for PCR analysis. Animals were kept in a room with 12 h light-dark cycle at 22°C, fed by OVA-free diet Altromin 1410 sterilized by irradiation and water *ad libitum*. Water was sterilized by autoclaving for GF and Lp-colonized mice. This study was carried out in accordance with the recommendations of the Committee for the Protection and Use of Experimental Animals of the Institute of Microbiology Academy of Sciences of the Czech Republic. All protocols were approved by the same committee.

### Experimental Protocol

**Figure 1:**
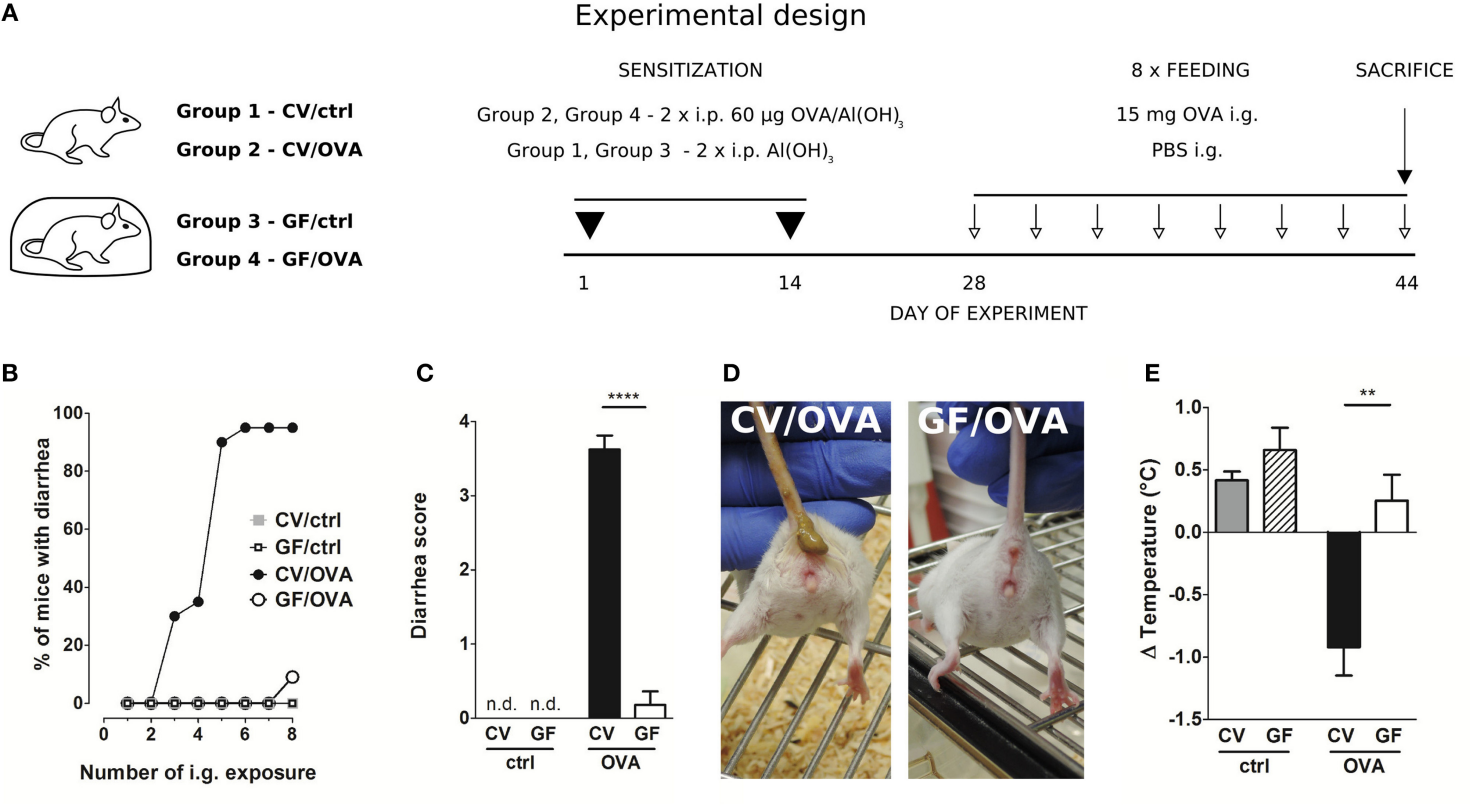
Germ-free mice fail to develop symptoms of OVA-induced food allergy. **(A)** Experimental design: conventional (CV) and germ-free (GF) mice were sensitized twice within a two-week interval by intraperitoneal (i.p.) injection of 60 μg OVA in Al(OH)_3_ (alum) followed by intragastric gavage (i.g.) with 15 mg OVA in PBS 8 times within 17 days (OVA groups). Age-matched control mice were injected with PBS/alum and gavaged by PBS (ctrl groups). After the last i.g. exposure, rectal temperature was assessed and mice were sacrificed by cervical dislocation. Sera and small intestine samples were collected for further analysis. Graphic art was created in freely available professional vector graphics editor Inkscape 0.92 (https://inkscape.org/). **(B)** The occurrence of diarrhea was monitored for 60 min after each i.g. administration in control CV (gray squares), control GF (open squares), in OVA-treated CV (black circles), and OVA-treated GF (open circles) mice. **(C)** The diarrhea score was assessed according to the scoring method described in Material and Methods section. **(D)** Representative pictures of CV/OVA and GF/OVA mice after the last i.g. OVA exposure. **(E)** The rectal temperature was measured 30 min after the last i.g. exposure at day 44 of the experiment in CV/ctrl (gray bars), and GF/ctrl (dashed bars) control mice, and in CV/OVA (black bars) and GF/OVA (white bars) OVA-treated mice. Difference in the temperature before and after challenge is shown. Data are plotted as mean values ± SEM. Pooled values of at least two independent experiments (CV/ctrl *n* = 8, CV/OVA *n* = 14, GF/ctrl *n* = 9, GF/OVA *n* = 11 mice per group) are shown. ^**^P ≤ 0.01, ^****^P ≤ 0.0001.

Female 8-week-old CV, GF, exGF and Lp mice were sensitized *i.p*. within two-week interval with 60 μg of OVA (Worthington, USA) together with 100 μl Alu-Gel-S (Serva, DE) adjuvant and PBS in a final volume of 200 μl on day 1 and 14. Control mice received 100 μl of PBS mixed with 100 μl of Alu-Gel-S. Two weeks after the second *i.p*. sensitization, mice were challenged 8 times at 2–3 day intervals (days 28–44) by *i.g*. gavages of 15 mg OVA in a final volume of 150 μl (OVA groups). Control mice received 150 μl of PBS by *i.g*. gavages (ctrl groups). Diarrhea occurrence was monitored for 30–60 min after each *i.g*. exposure. The diarrhea score was assessed according to the following criteria: 0–normal, well-formed stool, 1–soft, sticky well-formed stool, 2–not formed stool, 3–liquid diarrhea, 4–more than two episodes of liquid diarrhea after the antigen gavage during the treatment period. The temperature was measured by Thermocouple Thermometer with mouse rectal probe (World Precision Instruments Inc., USA) 30 min after the last *i.g*. exposure (Figure 1A).

### Isolation of Bacterial DNA From Cecal Content and 16S rDNA PCR Amplification

Total DNA from 150 mg cecal content was isolated by ZR Fecal DNA kit according to manufacturer‘s instructions (Zymo Research, USA). The purity, integrity and concentration of nucleic acids were confirmed by agarose gel electrophoresis and UV spectrophotometry as previously described (34). Bacterial 16S rDNA was amplified using PCR with the universal primers 27F (5′ AGA GTT TGA TCC TGG CTC AG 3′) and 1492R (5′ GGT TAC CTT GTT ACG ACT T 3′) as previously described (35). Ten ng of chromosomal DNA from *Escherichia coli* was used as a positive control. Amplification products were separated by electrophoresis in 1.2% agarose gel, visualized using GelRed™ Nucleic Acid Gel Stain (Biotinum, USA) and images were obtained by Fluorescent Image Analyser FLA-7000 (Fujifilm Corporation, JP).

### Quantification of OVA-Specific Antibodies

Blood samples were collected at sacrifice and serum was collected after centrifugation. OVA-specific serum IgE, IgG1, IgG2a, and IgA levels were determined by ELISA (36). Briefly, 96-well-microtiter plates were coated with OVA (5 μg/ml). Serum samples were diluted 1/10 for IgE, 1/10,000 for IgG1, 1/100 for IgG2, and 1/10 for IgA. Rat anti-mouse IgE, IgG1, IgG2a, and IgA antibodies (1 μg/ml, Pharmingen, San Diego, CA, USA) were applied, followed by peroxidase-conjugated anti-rat IgG antibodies (1/1,000, Jackson, Immuno Labs, West Grove, PA, USA) for detection. Antibody levels were reported as optical density (OD). The activity of OVA-specific IgE in serum was measured by rat basophil leukemia (RBL) cells degranulation assay as described previously (36).

### Cellular Immune Response

At sacrifice, spleens were aseptically removed and single-cell suspensions were prepared in RPMI-1640 containing 10% fetal bovine serum (BioClot GmbH, Aidenbach, Germany) and 1% Antibiotic-Antimycotic solution (Sigma-Aldrich). Cells (6 × 10^5^/well) were cultured in a flat-bottom 96-well plate (TPP, Trasadingen, Switzerland) without any stimuli or in the presence of OVA (100 μg/well) for 72 h (37°C, 5% CO_2_). Supernatants were collected and stored at −40°C until analyses. Levels of IL-4, IL-5, IL-10, IL-13, and IFN-γ were determined by the MILLIPLEX MAP Mouse Cytokine/Chemokine Panel (Millipore, USA) according to manufacturer's instructions and analyzed with the Bio-Plex System (Bio-Rad Laboratories, USA). Values are reported in pg/ml after subtraction of baseline levels of non-stimulated cell cultures.

### ELISA for Mast Cell Protease-1 and Cytokines in Jejunal Homogenates

Jejunum was aseptically removed and homogenate was prepared as followed. Protease inhibitor (Roche, DE) supplemented with 0.5% Triton X (Sigma-Aldrich, USA) was added to jejunum samples in the ratio 9:1 (w/w). After cooling on ice, the jejunum was homogenized for 1 min/40 Hz using Tissue Lyzer and stainless steel beads 7 mm (Qiagen, DE), frozen in liquid nitrogen, thawed, and homogenized again. Supernatants were collected after centrifugation and stored at −80°C. Protein content of the homogenates was determined by the Pierce™ BCA Protein Assay Kit (ThermoFisher Scientific, USA) using albumin as a standard. Levels of mouse mast cell protease-1 (MCPT-1) in serum and jejunal homogenates was determined by commercial kit Ready-SET-Go!® (eBioscience, USA) according to manufacturer's instructions. Levels of IL-4, IL-13, and TNF-α in jejunal homogenates were measured by the MILLIPLEX MAP Mouse Cytokine/Chemokine Panel (Millipore, USA) according to manufacturer's instructions and analyzed with the Bio-Plex System (Bio-Rad Laboratories, USA). MCPT-1 and cytokine levels in jejunal homogenates are represented per 1 mg of total protein.

### Isolation of Peritoneal Cavity Cells and Small Intestine Lamina Propria Mononuclear Cells

Naive CV and GF BALB/c mice were euthanized by isofluran and peritoneal cavity lavage was performed twice with 5 ml of cold PBS containing 0.1% sodium azide and 0.2% gelatin from cold water fish skin (PBS-gel) (Sigma-Aldrich, USA). Small intestine mononuclear cells were isolated according to Scott et al. (37) with minor modifications. Briefly, small intestine was excised and washed in cold PBS. Fat tissue and Peyer's patches were removed. The intestine was cut into small pieces and washed by 3% RPMI (Sigma-Aldrich, 3% FCS, pen/strep and HEPES). The tissue was incubated in 30 ml warm 3% RPMI with 5 mM EDTA and 1 mM DTT for 20 min at 37°C on orbital shaker (200 rpm). After washing with RPMI with 5 mM EDTA and PBS, the tissue was minced with scissors, mixed with 10 ml of digest solution [RPMI, 200 mM glutamin, pen/strep, sodium pyruvate, non-essential amino acids, HEPES, 0.1 mg/ml of Liberase (Roche, Switzerland), and 0.5 mg/ml of DNase 1 (Roche, Switzerland)] and incubated 30 min at 37°C on orbital shaker (200 rpm). Ten milliliters of 3% RPMI was added to stop the digestion. The digested tissue was filtered through 70 μm and 40 μm filter and centrifuged at 450 × g for 5 min. Cells were resuspended in 1 ml of complete RPMI.

### Flow-Cytometry Analysis

Cells (10^6^/well) were blocked for 10 min at 4°C in dark by 20% rat heat-inactivated serum in PBS containing 0.1% sodium azide. Staining was performed with fluorochrome labeled anti-mouse monoclonal Abs: CD45-FITC (eBioscience, USA; clone 30-F11), FcεRIα-phycoerythrin (BioLegend, USA; clone MAR-1), and CD117(c-kit)-APC-eFluor® 780 (eBioscience, USA; clone 2B8) according to the manufacturer's recommendation. After 30 min cells were washed four times by cold PBS-gel and data were acquired by FACSCalibur (BD Immunocytometry Systems, Mountain View, CA) or FACS Aria III (BD Immunocytometry Systems, Mountain View, CA) flow cytometer. Analysis was performed using FlowJo software (Tree Star, Ashland, OR, USA).

### Cutaneous Activation of Mast Cells

Intraplantar injection was performed with compound 48/80 (Sigma-Aldrich, USA; 0.5 μg/10 μl per paw) or PBS alone (10 μl per paw) into hind footpad of naive GF and CV mice. Changes in paw width were measured with digital caliper (±0.01 mm; Festa, Czech Republic) at time 0, 30, and 60 min. The baseline paw widths (time 0) for each mouse were measured immediately after injection and subtracted from paw widths after application to calculate tissue edema.

### Histology

Intestinal tissue specimens were fixed with 4% paraformaldehyde for 24 h followed by storage in 80% ethanol. Right ears were fixed in Carnoy's fluid for 30 min and transferred to 96% ethanol. Collected and fixed tissue specimens were dehydrated by using increasing concentrations of ethanol and transferred into methyl salicylate, benzene, benzene-paraffin and paraffin. Sections (5 μm) were deparaffinized in xylene and rehydrated through an ethanol to water gradient and stained for chloracetate esterase activity which is characteristic for mast cell granula. Reagent solution was prepared by mixing of 4% pararosaniline, 2 mol/l HCl, 4% aqueous sodium nitrite, 0.07 mol/l phosphate buffer (pH 6.5), and substrate solution (Naphthol AS-D chloroacetate dissolved in N-dimethylformamide) (all Sigma-Aldrich, USA). The sections were stained by reagent solution for 30 min in the dark and counterstained with hematoxylin for 2 min. The numbers of mast cells per randomly selected villi in jejunum and per 0.1 mm^2^ in the ear tissue were determined.

### RNA Isolation and Real-Time PCR

Jejunal tissues were stored in RNA-later reagent (Sigma-Aldrich, USA) overnight at 4°C and kept at −80°C until processed. Tissue samples were homogenized by Precellys 24 tissue homogenizer (Bertin Technologies, FR) at 5,000 rpm for 20 s using tubes with zirconium oxide beads. RNA was isolated via RNeasy Mini kit (Qiagen, Valencia, CA). An iScript cDNA Synthesis Kit (BioRad Laboratories, USA) was used to generate cDNA. Real-Time (RT) PCR was performed on the LightCycler® 480 instrument (Roche, DE) using LightCycler® 480 SYBR Green I Master according to the manufacturer's instructions (Roche, DE). β-actin was used as an internal control to normalize gene expression using the 2–ΔCt method (38). RT PCR primer sequences are listed in Supplementary Table 1.

### Statistical Analysis

Statistical analysis between multiple groups was performed by ANOVA with Tukey's multiple comparison test. Differences between two groups were evaluated using *t*-test. GraphPad Software was used to evaluate the data (GraphPad Prism 5.04, USA); *P* < 0.05 were considered significant. Data are expressed as means ±SEM.

## Results

### Germ-Free Mice Exhibit Reduced Susceptibility to Experimental Food Allergy

After intraperitoneal injection of OVA adsorbed to alum, CV and GF mice were challenged by oral gavage of OVA eight times over the period of 2 weeks (Figure 1A). Sensitized CV mice were most likely to develop experimental FA after five OVA challenges, with ~90% of these animals exhibiting allergic diarrhea (Figure 1B). In contrast, none of the GF mice developed diarrhea at this stage. Only after the eighth dose was diarrhea detected in 10% of GF animals (Figure 1B). This is reflected in the allergic diarrhea score where the majority of sensitized CV animals exhibited more than two episodes of liquid diarrhea after antigen gavage during the treatment period (Figures 1C,D). Sensitized CV mice also exhibited reduced core body temperature after the eighth OVA challenge. This was not observed in the GF animals (Figure 1E).

### High Levels of Th2-Accociated Specific Serum Antibodies and Spleen Cytokines Do Not Correlate to the Low Susceptibility of Germ-Free Mice to Develop Food Allergy

**Figure 2:**
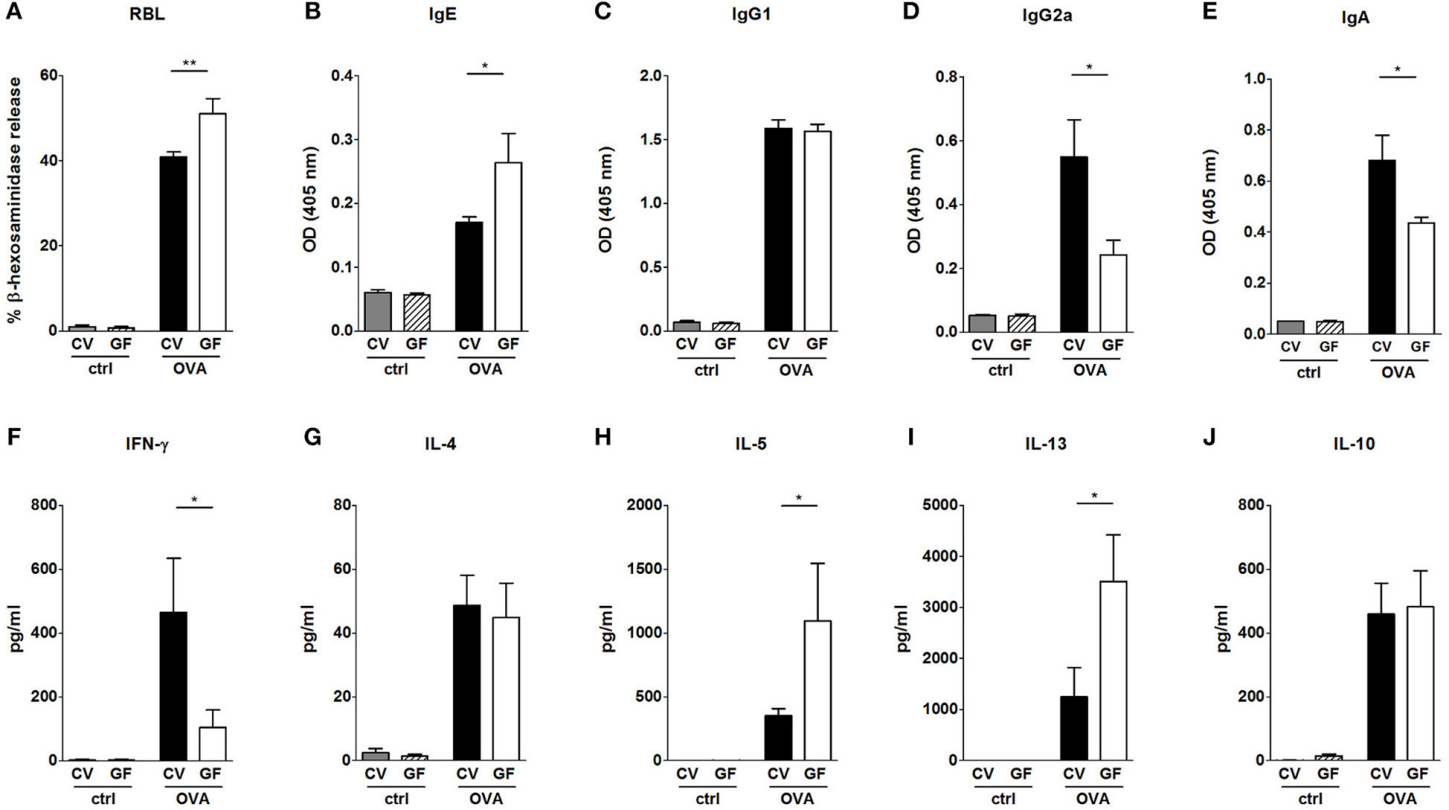
Impact of bacterial colonization on the levels of OVA-specific antibodies in sera and on OVA-induced cytokine production in splenocytes. Levels of OVA-specific antibodies were measured in sera of control CV (gray bars) and GF (dashed bars) mice, and sera of OVA-treated CV (black bars), and GF (white bars) mice. **(A)** Functional IgE in serum was measured by OVA-mediated β-hexosaminidase release from rat basophil leukemia cells (RBL). OVA-specific **(B)** IgE, **(C)**, IgG1, **(D)**, IgG2a, and **(E)**, IgA were measured by ELISA and expressed as optical density (OD). Levels of **(F)**, IFN-γ, **(G)**, IL-4, **(H)**, IL-5, **(I)**, IL-13, and **(J)**, IL-10 in splenocyte culture supernatants were measured by MILLIPLEX Cytokine panel. Cytokine levels are expressed after subtraction of base line levels of unstimulated splenocytes. Data are plotted as mean values ± SEM. Pooled values of at least two independent experiments (CV/ctrl *n* = 8, CV/OVA *n* = 14, GF/ctrl *n* = 9, GF/OVA *n* = 11 mice per group) are shown. ^*^P ≤ 0.05, ^**^P ≤ 0.01.

Systemic allergic sensitization and oral challenge with OVA led to the induction of OVA-specific IgE, IgG1, IgG2a, and IgA in sera in both experimental groups (Figures 2A–E). In agreement with previous studies (10, 12, 35), sensitized GF mice exhibited increased levels of OVA-specific IgE in comparison to CV animals (Figures 2A,B). The functionality of the OVA-specific IgE was tested in a rat basophil leukemia cell degranulation assay. Sera from sensitized and challenged GF animals induced higher levels of β-hexosaminidase release in comparison to sera from CV mice (Figure 2A). While levels of IgG1 were comparable between CV and GF mice (Figure 2C), higher specific IgG2a (Figure 2D) and IgA (Figure 2E) levels were detected in mice raised in the presence of microbiota compared to GF animals. Systemic allergen-specific cellular responses were evaluated by re-stimulating splenocytes with OVA *ex vivo* (Figures 2F–J). Stimulation of cells derived from sensitized and challenged GF mice led to the induction of Th2 cytokines (Figures 2G–J). Levels of IL-5 and IL-13 were significantly higher in GF mice compared to CV mice (Figures 2H,I). A lack of bacterial exposure was associated with reduced levels of OVA-specific IFN-γ production (Figure 2F).

### Absence of Microbial Colonization Leads to a Lower Density of Mast Cells in the Gut and Reduced Levels of Local and Systemic MCPT-1

**Figure 3:**
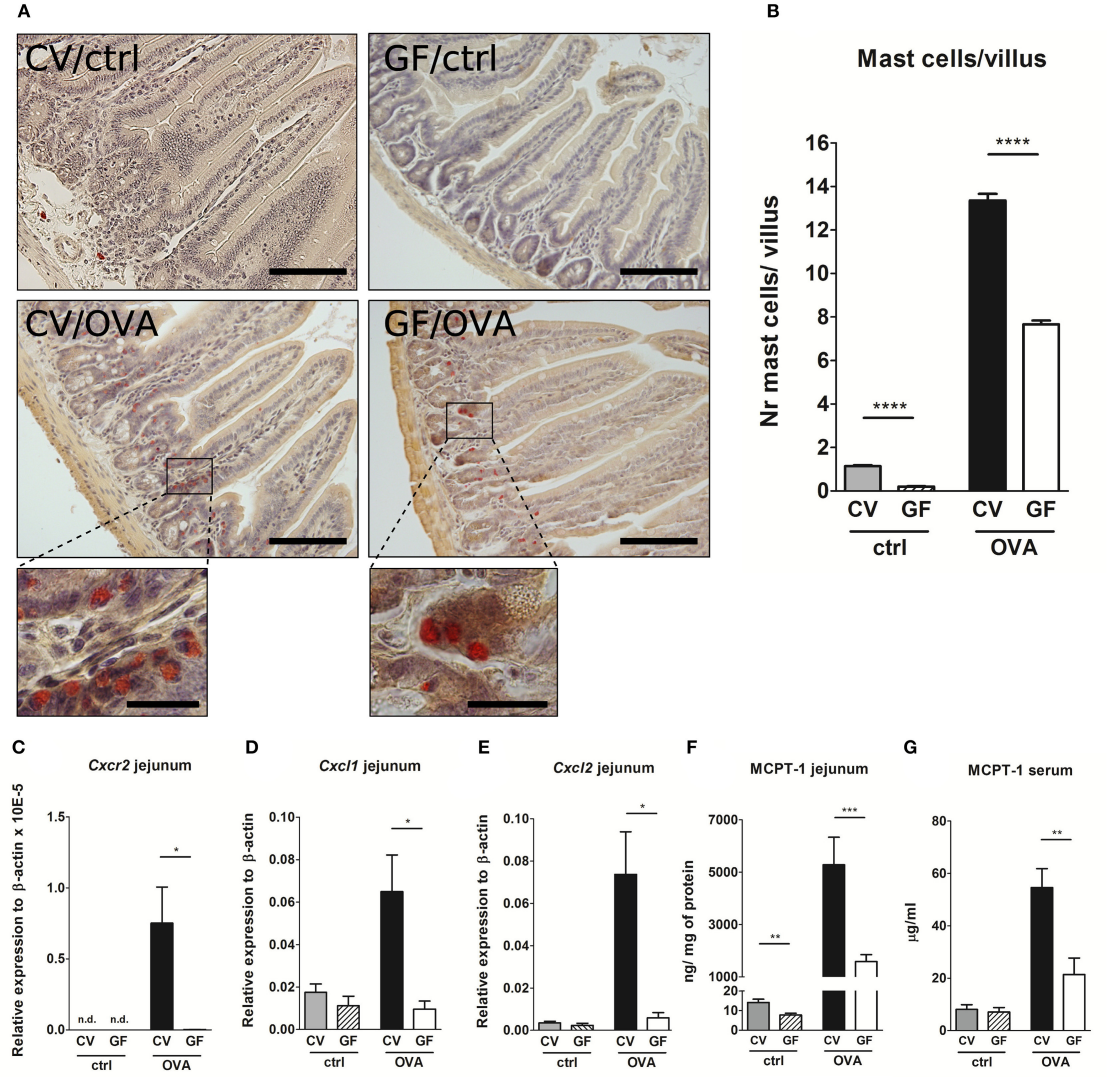
Germ-free mice exhibit low numbers of intestinal mast cells, low expression of *Cxcr2* and its ligands, and low levels of Mast cell protease-1 after OVA-sensitization and challenge. **(A)** Histological staining of jejunal sections for mastocytosis by hematoxylin/pararosaniline was performed on samples from control conventional (CV/ctrl) and germ-free (GF/ctrl), and from OVA-treated CV (CV/OVA) and germ-free (GF/OVA) mice (scale bars, 100 μm; inset scale bars, 25 μm) **(B)**, Quantification of mast cells per villus in jejunal sections (CV/ctrl *n* = 3, CV/OVA *n* = 8, GF/ctrl *n* = 5, GF/OVA *n* = 5 mice per group). Messenger RNA expression of **(C)**, *Cxcr2* **(D)**, *Cxcl1*, and **(E)**, *Cxcl2* (in the jejunal tissues was determined by real-time PCR. Relative expression to β-actin is shown (CV/ctrl *n* = 6, CV/OVA *n* = 7, GF/ctrl *n* = 6, GF/OVA *n* = 5 mice per group). Mast cell protease-1 (MCPT-1) levels were determined in **(F)**, jejunal homogenates and in **(G)**, sera of control conventional (CV; gray bars) and germ-free (GF; dashed bars) mice, and in OVA-treated CV (black bars), and GF (white bars) mice by ELISA. Data are plotted as mean values ± SEM. Pooled values of at least two independent experiments (CV/ctrl *n* = 8, CV/OVA *n* = 14, GF/ctrl *n* = 9, GF/OVA *n* = 11 mice per group) are shown. ^*^P ≤ 0.05, ^**^P ≤ 0.01, ^***^P ≤ 0.001, ^****^P ≤ 0.0001.

Given the fact that GF mice were protected from the development of food allergy despite their ability to produce large amounts of OVA-specific IgG1 and IgE as well as pro-allergic systemic cellular responses, we hypothesized that the lack of microbial stimulation resulted in a non-functional effector compartment in the gut. It has been well-established that gastrointestinal symptoms during oral antigen-induced anaphylaxis depend not only on IgE but also on the numbers of MC in the intestine (30). Here we show that sham-treated GF mice displayed significantly lower numbers of mucosal MC compared to CV mice (Figures 3A,B), accompanied by the lower levels of MCPT-1 in the intestinal tissue (Figure 3F). This phenomenon, although not as pronounced, was also confirmed in the skin where GF mice harbored 20% fewer MC in comparison to CV mice (Figures S1A,B). Although the numbers of intestinal MC increased both in CV and GF mice after OVA-treatment, the numbers were significantly lower in GF mice compared to CV mice (Figures 3A,B). This was associated with lower expression of intestinal CXCR2 and its ligands CXCL1 and CXCL2, markers associated with the recruitment of MC to the gut (Figures 3C–E). Concomitantly, the local production of Th2-associated IL-4 and IL-13, and pro-inflammatory TNF-α was significantly lower in the jejunum of sensitized and challenged GF mice compared to CV animals (Figures S2A–F). Levels of MCPT-1 in the gut and in serum were significantly lower in OVA-treated GF mice compared to CV mice (Figures 3F,G). Although the numbers of intestinal MC in OVA-treated GF mice reached ~60% of those detected in CV mice, the production of MCPT-1 in the jejunum reached only 30% of the levels detected in OVA-treated CV mice. These observations suggest that also the maturation status and functionality of MC are influenced by microbiota.

### Lack of Microbial Colonization Leads to Reduced Expression of Mast Cells Expansion, Differentiation, and Survival Factor Associated With Reduced Mast Cells Degranulation *in vivo*

**Figure 4:**
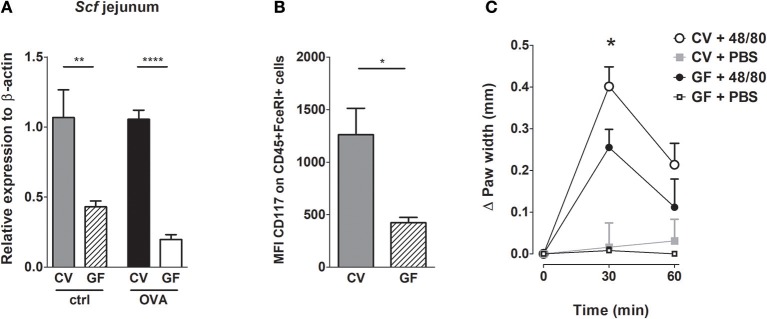
Germ-free mice have less mature mast cells and these cells are functionally impaired. **(A)** Messenger RNA expression of Stem cell factor (*Scf*) in the jejunal tissues was determined by real-time PCR. Relative expression to β-actin is shown. CV/ctrl (gray bars) *n* = 5, GF/ctrl (dashed bars) *n* = 6, CV/OVA (black bars) *n* = 5, GF/OVA (white bars) *n* = 5 mice per group. **(B)** Mean fluorescence intensity of CD117 was determined by flow cytometry on CD45+FcεRIα+ cells isolated from small intestine. CV (gray bars) *n* = 3, GF (dashed bars) *n* = 3 mice per group. **(C)** GF and CV mice were injected with degranulation-inducing compound 48/80 or PBS and edema was recorded. Data are presented as paw width after subtraction of baseline values, GF 48/80 (black circles) *n* = 6, CV 48/80 (open circles) *n* = 8, GF PBS (open squares) *n* = 5, CV PBS (gray squares) *n* = 8 mice per group. Data are plotted as mean values ± SEM. ^*^P ≤ 0.05, ^**^P ≤ 0.01, and ^****^P ≤ 0.0001.

Stem cell factor (SCF) is a key factor in MC biology (39). Besides being the major chemotactic factor, SCF is indispensable for MC proliferation and for their survival, differentiation and maturation (24). We therefore determined the SCF expression in the small intestinal tissue. We found that SCF expression was significantly lower in GF mice in comparison CV animals, both in naïve controls and OVA-treated mice (Figure 4A). We further assessed the expression of the SCF receptor on the intestinal MC in naïve GF and CV mice by flow-cytometry (gating strategy in Figure S3). In the absence of microbiota, the levels of mean fluorescence of surface CD117 were significantly lower on GF MC compared to MC isolated from CV intestines, suggesting their lower maturation status (Figure 4B). Furthermore, GF mice exhibit significantly lower numbers of mature and significantly higher numbers of immature MC compared to CV mice as assessed by the determination of the peritoneal FcεRIα^+^CD117^+^ granularity by FACS (Figures S4A–C). To functionaly confirm the role of the microbiota in MC maturation and function, we injected footpads of GF and CV mice with the compound 48/80, which has been used to induce MC degranulation and is associated with tissue edema *in vivo* (40). We clearly showed that injection of the 48/80 induced edema in footpad of CV mice and this was significantly less pronounced in GF animals (Figure 4C).

### Reconstitution of Germ-Free Mice With Complex Microbiota but Not With a Single Bacterial Strain Restores the Susceptibility to Food Allergy

**Figure 5:**
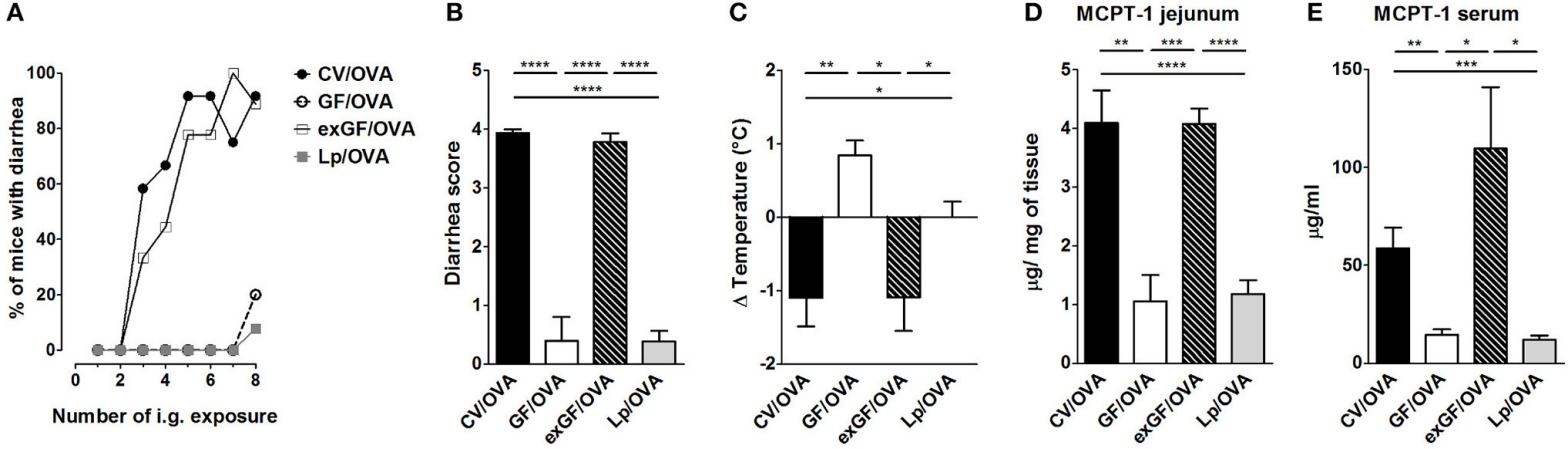
Colonization of germ-free mice by the conventional microbiota but not by single bacterial strain *Lactobacillus plantarum* WCFS1 restores sensitivity to OVA-induced food allergy. Germ-free (GF) mice were colonized by co-housing with age-matched conventional (CV) animals (exGF group) or mono-associated with the strain *L. plantarum* WCFS1 (Lp group). At the age of 8 weeks, all groups were submitted to OVA-sensitization and challenge, as described in Figure 1. **(A)** The occurrence of diarrhea during OVA treatment. **(B)** The diarrhea score for each group was assessed according to the scoring described in section Materials and Methods section. **(C)** The rectal temperature was taken 30 min after the last i.g. exposure at day 44. Difference in the temperature before and after challenge is shown. Mast cell protease-1 (MCPT-1) levels were determined in jejunal homogenates **(D)**, and in sera **(E)**, by ELISA. Data are plotted as mean values ± SEM. CV/OVA (*n* = 8), GF/OVA (*n* = 5) and pooled values of two independent experiments for exGF/OVA (*n* = 9) and Lp/OVA (*n* = 13) colonized mice are shown. ^*^P ≤ 0.05, ^**^P ≤ 0.01, ^***^P ≤ 0.01, ^****^P ≤ 0.0001.

To test whether the susceptibility to food allergy can be restored, we colonized GF mice by co-housing them with CV mice or by gavaging them with human *Lactobacillus* isolate *L. plantarum* WCFS1. Successful conventionalization was verified by decrease in cecal weight and the presence of bacterial DNA measured by PCR in cecal samples (Figures S5A,B). In conventionalized mice (exGF), the incidence of diarrhea, the diarrhea score and the degree of hypothermia reached levels comparable to those observed in CV mice (Figures 5A–C). Furthermore, these mice exhibited higher levels of MCPT-1 in the jejunum and serum (Figures 5D,E). In contrast to conventionalized ex-GF mice, GF mice mono-colonized with bacterial strain *L. plantarum* failed to develop clinical symptoms of experimental FA (Figures 5A–C). There was no significant difference in the occurrence of diarrhea, diarrhea severity or in the level of hypothermia between Lp mono-colonized mice and GF animals. Moreover, *L. plantarum* did not restore the production of MCPT-1 in the jejunum and serum to those observed in CV mice; levels were similar to those observed in GF mice (Figures 5D,E).

## Discussion

Here we report that despite showing high levels of allergic sensitization, GF BALB/c mice are protected against the development of OVA-induced FA, exhibiting low incidence of diarrhea and hypothermia. In the intestine, GF mice displayed reduced numbers of MC and lower levels of MCPT-1 after allergic challenge in comparison to CV mice. Further we confirmed the altered MC maturation and function in the absence of microbiota. Finally, colonization of GF mice with conventional microbiota, but not their mono-association with single bacterial strain *L. plantarum* WCFS1, induced the susceptibility of exGF mice to OVA-induced FA.

In accordance with the hygiene hypothesis, GF animals have been shown to develop increased levels of serum IgE after allergic sensitization in comparison to their CV counterparts (9–12). Here we show that sensitization and challenge lead to increased OVA-specific IgE levels in sera and increased production of OVA-specific IL-5 and IL-13 in re-stimulated splenocytes from GF mice compared to CV animals. The concept that signals derived from commensal bacteria are crucial to normalize elevated Th2 responses have been tested previously by us and others. For example, colonization of GF mice with conventional or SPF microbiota, with a well-defined mixture of different strains, or with only a single bacterial strain led to reduced levels of serum IgE and Th2 cytokine production in comparison to GF animals (11, 12, 31, 41). Recently, we have expanded this observation to residual bacterial fragments and showed that the presence of LPS in sterile food was sufficient to reduce high levels of allergen-specific IgE in GF sensitized mice (35).

Surprisingly, despite the elevated systemic OVA-specific humoral and cellular Th2 responses, GF mice were protected from FA symptoms, i.e., they failed to develop allergic diarrhea and hypothermia. This observation is in disagreement with studies by Cahenzli et al., Rodriguez et al. and Stefka et al. who showed that GF mice are more susceptible to FA (10, 31, 42). There are several possible explanations for this discrepancy. First, differences in the sensitization and challenge protocols. We used 2 intraperitoneal doses of 60 μg OVA/Alum followed by eight challenges of 15 mg OVA by gavage, as previously established by Golias et al. (36). In contrast, Cahenzli et al. sensitized mice with one subcutaneous dose of 50 μg OVA/Alum followed by single oral challenge with 50 mg OVA (31). A very different model was used by Stefka et al. and Rodriguez et al., where mice were sensitized by intragastric gavages with antigen admixed with cholera toxin (CT) (10, 42). CT is known as a potent mucosal adjuvant which induces mobilization and maturation of intestinal immune cells (43) as well as the induction of Th1/Th2/Th17 responses (44). Thus, the application of CT to GF mice might provide signals for maturation or recruitment of MC to the gut tissue. Second, there are differences in the readouts and reporting of clinical symptoms. In Cahenzli et al. and in Stefka et al. the allergic sensitization and oral challenge did not induce hypothermia in SPF or CV mice (10, 31). This is surprising since we and others have shown that immunization in the presence of adjuvant followed by oral challenge with or without adjuvant results in clinical symptoms, such as hypothermia or diarrhea in both conventional animals or animals with SPF microbiota (2, 28, 30, 45, 46). Third, there are differencies in sterilization methods of chow for GF animals. In our study, GF animals were fed by γ-irradiated chow, which is in contrast to sterilization method used by Cahenzli et al. where the chow was autoclaved (31). Autoclaving leads to formation of advanced glycation end-products as a result of chemical reaction between reducing sugars and amino acids in proteins or lipides (Meillard reaction) (47). These products has been recently pointed at as one of the possible causes of increasing FA incidence in Western countries (48). This is of special interest, as autoclaved, but not irradiated food has been shown to increase the numbers of intestinal MC in GF rats (49).

To determine the discrepancy between high levels of sensitization and the absence of FA symptoms, we investigated the presence of intestinal MC, which are widely recognized key effectors of allergy in the periphery (27, 28, 46). Here we demonstrate that GF animals exhibit low levels of MC in the gut under homeostatic conditions and that MC numbers are still reduced after allergen sensitization and oral challenge in comparison to CV mice. The reduced numbers of intestinal MC in GF mice were accompanied by decreased expression of *Cxcr2* and its ligands in intestinal tissue. Concomitantly, challenged GF mice had significantly lower production of MCPT-1 both locally in the intestinal tissue and systemically in sera. Further experiments are required to dissect whether the microbiota impacts MC recruitment and/or function directly or indicrectly via the action on intestinal epithelial or innate lymphoid cells.

Together with a previous report about the crucial role of MC for the development of FA symptoms (27), our data suggests that the MC homing to the intestinal effector compartment is impaired in GF animals. Chen et al. have also shown that the severity of FA correlates with intestinal MC numbers as mouse strains without intestinal MC (i.g. C57BL/6 or C3H/HeJ) did not exhibit clinical symptoms of experimental FA after antigen sensitization and challenge (28). However, due to the different mouse strains used in the study by Chen et al. were not littermate controlled, the role of microbiota in the susceptibility to food allergy cannot be ruled out (28).

In our model, intestinal MC numbers in sensitized and challenged GF animals reached 60% of those observed in CV animals. Yet the GF mice were fully protected from the development of allergic diarrhea and hypothermia. This indicates that not only the numbers of local MC in GF mice, but also the functionality and maturation status of MC are impaired in these mice. In a recent paper, Wang et al. clearly demonstrated that the microbiota drives the recruitment and functionality of skin MC through the LTA-TLR2-dependent production of SCF by keratinocytes (25). In agreement with this study, we have shown that the SCF expression was significantly lower in GF mice compared to the CV mice, both in naïve controls and OVA-treated groups. The low expression of intestinal SCF in GF mice was associated with reduced expression of CD117 on FcεRIα+ intestinal MC in these animals. Next, we addressed the impact of the microbiota on maturation status of intraperitoneal MC. Previously Dahlin et al. showed that the low granularity of MC corresponds with their low maturation status (50). We found that there was an increased percentage of MC with low granularity in GF animals compared to CV animals and that these immature MC expressed lower levels of CD117 (data not shown), a known surface maturation marker (25). These data confirmed our hypothesis that GF mice have low MC numbers in the intestine exhibiting lower maturation status.

In order to test the functionality of MC we challenged the GF and CV mice by injection of degranulation compound 48/80. GF mice exhibited significantly lower swelling compared to CV animals confirming the impaired functionality and lower maturation of MC in the absence of microbiota.

Finally, we could show that conventionalization rendered exGF mice sensitive to FA, as demonstrated by hypothermia, diarrhea, and elevated levels of MCPT-1 in the gut and serum. Interestingly, mice mono-colonized with Gram-positive strain *L. plantarum* remained unresponsive to OVA challenge in contrary to CV mice. This observation is surprising, as *L. plantarum* WCFS1 is a bacterial strain with strong immunomodulatory properties (15, 17, 51) and oral application of this strain aggravated the severity of peanut FA in a mouse model (14). Nevertheless, it has been well-documented that different bacterial strains, and even the strains of the same species, may differ in their immunomodulatory potential (52, 53). Whether mono-colonization by other bacterial strains (e.g., Gram-negative), or supplementing the GF mice with specific bacterial products (e.g., LPS, peptidoglycan, lipoteichoic acid) can impact the maturation and function of intestinal MC and render the mice susceptible to food allergy remains to be determined.

Taken together, we report here that commensal bacteria impact MC migration and maturation in the intestine, thus playing a key role in the susceptibility to food-induced allergy. Our model based on CT-free systemic sensitization and oral challenge leads to full development of hypothermia and diarrhea in CV settings but not GF mice, represents an important tool to investigate the role of microbiota in the development or prevention of infectious or immune-mediated inflammatory diseases. The mechanistic insight into the role of the commensals-MC-FA axis, with a focus on the microbiota-induced recruitment and maturation of MC in the intestinal mucosa, can pave the way to the design of novel strategies for the prevention and treatment of food allergy in humans.

## Author Contributions

MSch, LT, HK, and IS conceived and designed the experiments. MSch, PH, JG, CZ, TH, DS, and JA performed the experiments. MSch, PH, JG, CZ, TH, MS and DS analyzed the data. MSch, PH, UW, LT, HK, and IS wrote the paper. All authors reviewed the manuscript.

### Conflict of Interest Statement

The authors declare that the research was conducted in the absence of any commercial or financial relationships that could be construed as a potential conflict of interest.

## Abbreviations

CV: Conventional
ExGF: GF mice co-housed with conventional mice
FA: Food allergy
GF: Germ-free
IL: Interleukin
LPS: Lipopolysaccharide
MC: Mast cells
MCPT-1: Mast cell protease-1
OVA: Ovalbumin
PCR: Polymerase chain reaction
SCF: Stem cell factor
SPF: Specific pathogen free.
