# Supplementary material for: Germ-Free Mice Exhibit Mast Cells With Impaired Functionality and Gut Homing and Do Not Develop Food Allergy
Source: Front Immunol. 2019 Feb 12;10:205. doi: 10.3389/fimmu.2019.00205 (PMC6379318; doi:10.3389/fimmu.2019.00205)
Supplement: Supplementary file 1 [file Data_Sheet_1.pdf]

Supplementary Material

Table S1. Sequences of real-time PCR primers

Gene	Forward primer	Reverse primer
Cxcr2	ACTCCTTGGTGATGCTGGTC	CACAGGGTTGAGCCAAAAGT
Cxcl1	CGCTGCTGCTGCTGGCCACCA	GCTATGACTTCGGTTTGGGTGCAG
Cxcl2	AGTGAACTGCGCTGTCAATGC	AGGCAAACTTTTTGACCGCC
Il4	TCAACCCCCAGCTAGTTGTC	TGTTCTTCGTTGCTGTGAGG
Il13	CAGCATGGTATGGAGTGTGG	TGGGCTACTTCGATTTTGGT
Tnfα	TGCCTATGTCTCAGCCTCTTC	TTGTGAGTGTGAGGGTCTGG
Scf	AGCTTGACTACTCTTCTGGACA	TGGCCTCTTCGGAGATTCTTTT

Figure S1

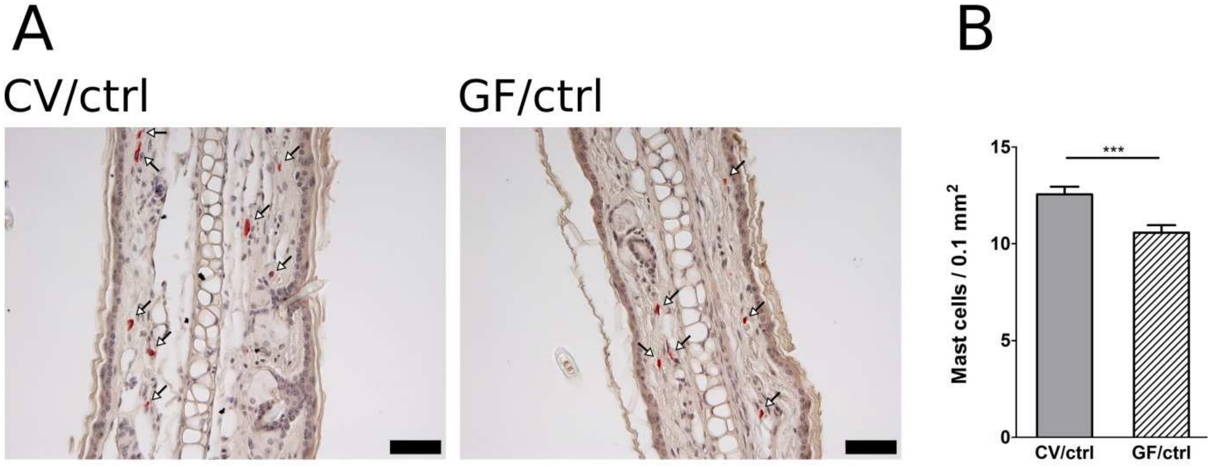

Figure S1. Germ-free mice exhibit reduced numbers of skin mast cells compared to conventional mice. A, Histological staining of ear sections for mastocytosis by hematoxylin/ pararosaniline was performed on samples from control conventional (CV/ctrl) and germ-free (GF/ctrl) animals, (scale bars, 50 µm). **B,** Mast cells were quantified per 0.1 mm2 area of the tissue. Pooled values of n = 5 mice per group are shown. The occurrence of mast cells in 8-12 independent areas were counted for each individual mouse. ***P ≤ 0.001.

Figure S2

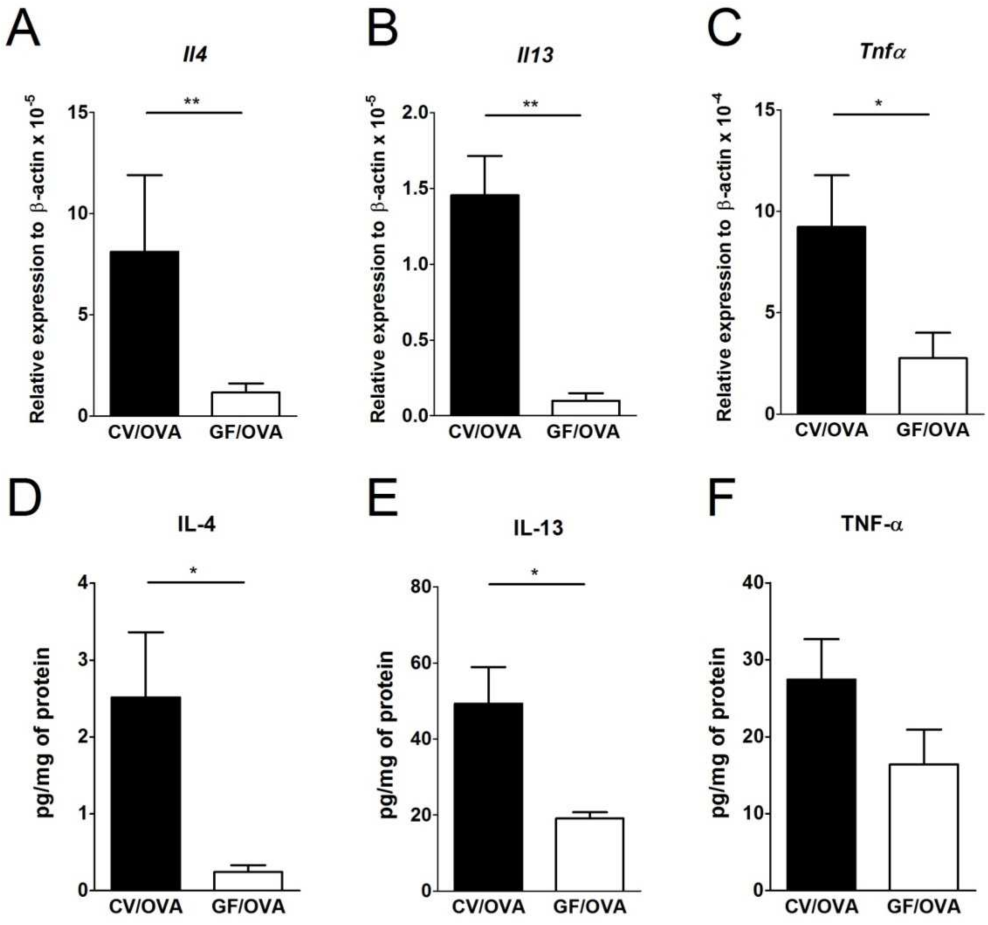

Figure S2. Messenger RNA and protein levels of IL-4, IL-13 and TNF-α are low in the jejunum of OVA-sensitized and challenged germ-free mice. A, Messenger RNA expression of *Il4*, **B,** *Il13* and **C,** *Tnfα* (in the jejunal tissues of OVA-treated conventional (CV/OVA; black bars; n = 7) and germ-free (GF/OVA; white bars; n = 5) mice was determined by Real-Time PCR. Relative expression to β-actin is shown. Protein levels of IL-4 (**D**), IL-13 (**E**) and TNF-α (**F**) in the jejunal tissue homogenates of OVA-treated CV (black bars, n = 14) and GF (white bars, n = 11) mice were determined by ELISA. Levels are normalized per 1 mg of protein. Data are plotted as mean values ± SEM. $^*P \leq 0.05$, $^{**}P \leq 0.01$.

Figure S3

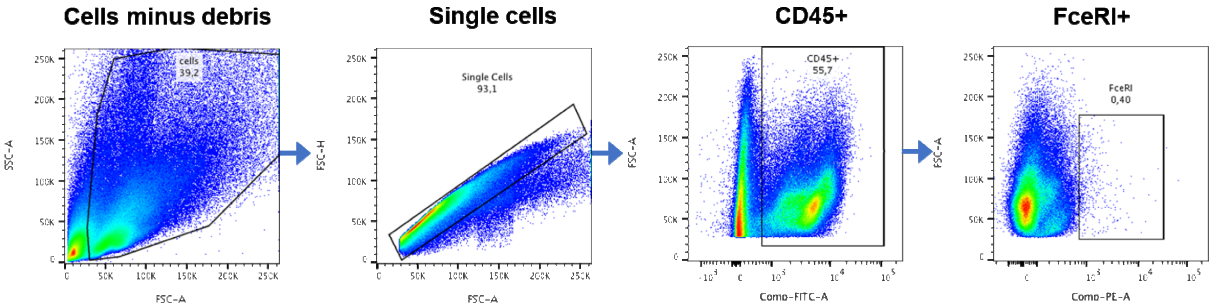

Figure S3. Gating strategy for CD45+FcεRIα+ population isolated from small intestine.

Figure S4

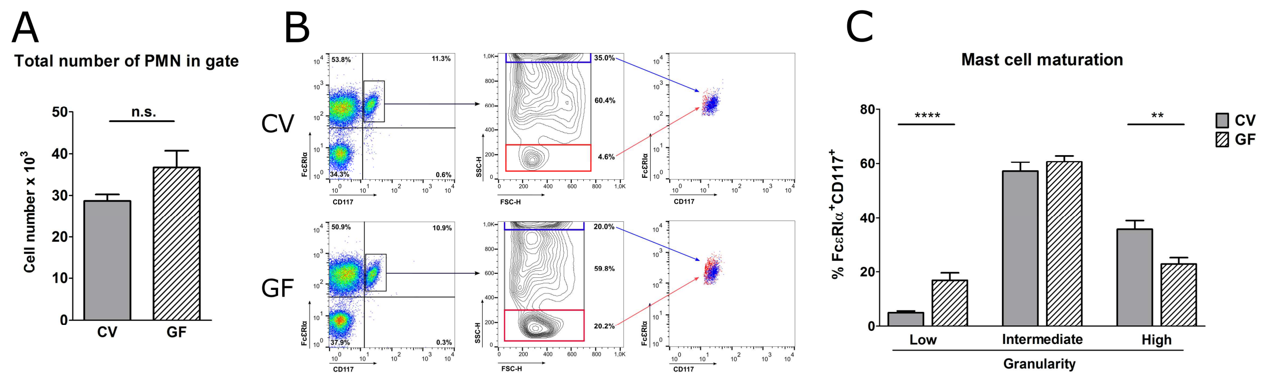

Figure S4. Flow cytometry analysis of peritoneal mast cells. A, Comparison of total numbers of gated polymorphonuclear (PMN) cells from intraperitoneal lavages from CV (n = 9) and GF (n = 9) mice. **B,** Representative flow cytometry analysis of cells from intraperitoneal lavages from CV and GF mice. Size (forward scatter - FSC) and granularity (side scatter - SSC) of FcεRIα and CD117 (ckit) positive cells (mast cells) out of total polymorphonuclear leukocytes were analyzed and displayed using counter plot. Three populations (low, intermediate and high) were established according to cell granularity and the percentage of their density was analyzed. **C,** FcεRIα$^+$CD117$^+$ polymorphonuclear cells from intraperitoneal lavage were gated and their maturation was assessed according to their granularity. CV (grey bars) n = 9, GF (dashed bars) n = 9 mice per group. Data are plotted as mean values ± SEM. $*P \leq 0.05$, $**P \leq 0.01$, $****P \leq 0.0001$.

Figure S5

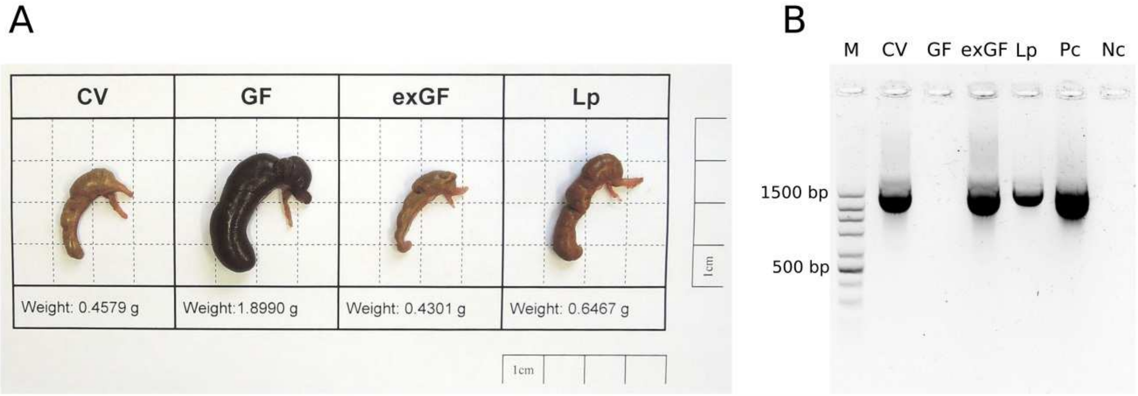

Figure S5: Successful colonization of germ-free mice with the conventional microbiota and with the *Lactobacillus plantarum* WCFS1. A, Representative ceca from conventional (CV), germ-free (GF), exGF (GF mice colonized by cohousing with age-matched CV animals) and *L. plantarum* WCFS1-monocolonized gnotobiotic (Lp) mice were dissected at the end of experiment, weighed and photographed. **B,** Total DNA from cecal content was isolated and PCR was performed with bacteria-specific primers. PCR products were separated by electrophoresis in 1.2% agarose gel. 10 ng of *E. coli* DNA was used as positive control (Pc), H2O as negative control (Nc), marker (M).
